# Interval Since Last HIV Test for Men and Women with Recent Risk for HIV Infection — United States, 2006–2016

**DOI:** 10.15585/mmwr.mm6724a2

**Published:** 2018-06-22

**Authors:** Marc A. Pitasi, Kevin P. Delaney, Emeka Oraka, Heather Bradley, Elizabeth A. DiNenno, John T. Brooks, Joseph Prejean

**Affiliations:** ^1^Division of HIV/AIDS Prevention, National Center for HIV/AIDS, Viral Hepatitis, STD, and TB Prevention, CDC; ^2^ICF International, Atlanta, GA.

Since 2006, CDC has recommended routine screening of all persons aged 13–64 years for human immunodeficiency virus (HIV) and at least annual rescreening of persons at higher risk (*1*). However, national surveillance data indicate that many persons at higher risk for HIV infection are not screened annually, and delays in diagnosis persist (*2*). CDC analyzed 2006–2016 data from the General Social Survey (GSS)* and estimated that only 39.6% of noninstitutionalized U.S. adults had ever tested for HIV. Among persons ever tested, the estimated median interval since last test was 1,080 days or almost 3 years. Only 62.2% of persons who reported HIV-related risk behaviors in the past 12 months were ever tested for HIV, and the median interval since last test in this group was 512 days (1.4 years). The percentage of persons ever tested and the interval since last test remained largely unchanged during 2006–2016. More frequent screening of persons with ongoing HIV risk is needed to achieve full implementation of CDC’s screening recommendations and to prevent new infections. Integration of routine screening as standard clinical practice through existing strategies, such as electronic medical record prompts (*3*), or through new, innovative strategies might be needed to increase repeat screening of persons with ongoing risk.

In 2006, CDC recommended one-time HIV screening of all persons aged 13–64 years and annual rescreening of persons at higher risk for HIV, including persons who inject drugs and their sex partners, persons who exchange sex for money or drugs, sex partners of HIV-infected persons, sexually active gay, bisexual, and other men who have sex with men (MSM), and heterosexual persons who themselves or whose sex partners have had more than one sex partner since their most recent HIV test (*1*). In 2017, CDC reiterated this annual screening recommendation for sexually active MSM based on a systematic literature review (*4*,*5*) that found that HIV incidence could be reduced significantly if MSM were screened annually (*6*,*7*). Despite this recommendation, a recent analysis of National HIV Surveillance System (NHSS) and National HIV Behavioral Surveillance (NHBS) data demonstrated that many persons at higher risk are not screened annually and that HIV diagnosis delays persist (*2*). Because NHSS data are based on reported diagnoses of HIV and do not include persons who test HIV-negative, and NHBS samples only persons at higher risk for HIV who reside in urban areas, these findings are not generalizable to the entire U.S. population (*2*). Population-based surveys such as the Behavioral Risk Factor Surveillance System (BRFSS) can be used to evaluate national HIV screening coverage, but BRFSS and most other population-based surveys lack sufficient information about HIV-related risk behaviors.

GSS is a biennial, household-based, multistage probability survey of noninstitutionalized U.S. adults aged ≥18 years that, since 2006, has included questions about HIV-related risk behaviors and HIV testing.^†^ During 2006–2016, overall survey response rates ranged from 61.3% to 71.4%.^§^ In this analysis, respondents were divided into four mutually exclusive HIV risk groups based on self-report of recent HIV-related risk behaviors: 1) men who had a male sex partner in the past 12 months; 2) men who did not have a male sex partner in the past 12 months but had multiple female sex partners, injected drugs, or paid or were paid for sex with a female sex partner in the past 12 months; 3) women who had multiple sex partners (male or female), injected drugs, or paid or were paid for sex in the past 12 months; and 4) respondents who did not have any of these risks. The first three groups were aggregated and categorized as persons with recent HIV risk. Data collected from the six biennial surveys were aggregated and used to estimate the weighted prevalence and 95% confidence interval (CI) of ever testing for HIV and the median number and interquartile range (IQR) of days since last test, stratified by demographics and HIV risk group. The median number of days since last test was also compared by survey year. Questions about HIV testing and risk behaviors were part of a computer-assisted self-interview module administered to a randomly selected subset of each survey sample. This analysis was limited to respondents who were asked if they were ever tested for HIV infection and provided a “yes” or “no” response. All estimates were weighted to account for the multistage sampling design.

Among 15,956 total respondents, 11,896 (74.6%) were asked if they had ever tested for HIV. Of these, 208 (1.7%) provided a response of “don’t know” or refused to answer, yielding an analytic sample of 11,688 respondents. Overall, 39.6% had ever tested, and the median estimated time since last test was 1,080 days or almost 3 years (Table 1). The percentages ever tested were highest among persons aged 25–34 years (54.4%) and 35–44 years (55.1%), non-Hispanic African American or black (black) persons (57.4%), and persons with recent HIV risk (62.2%). The median estimated number of days since last test was fewest among blacks (534 days), persons aged 18–24 years (332 days), persons with an annual household income of <$35,000 (767 days), and persons with recent HIV risk (512 days). Among persons with recent HIV risk, the median number of days exceeded 365 days (i.e., annual screening) in every survey year (Figure), and the percentage ever tested ranged from 60.0% to 66.7% across years (data not shown). The percentage ever tested was highest for men who had a male sex partner (71.0%) and women with HIV risk in the past 12 months (65.9%) (Table 2). Median interval since last test was shorter among persons in all three HIV risk groups (men who had a male sex partner [459 days], other at-risk men [610 days], and women [416 days]) compared with persons with no recent HIV risk (1,360 days).

**TABLE 1:** Percentage of persons ever tested for human immunodeficiency virus (HIV) infection and median number of days since last HIV test by demographic characteristics and recent HIV risk — General Social Survey, United States, 2006–2016

Characteristic	No.	Ever tested	Days since last test
		Weighted % (95% CI)	Median (IQR)
**Total**	**11,688**	**39.6 (38.4–40.8)**	**1,080 (325–3,023)**
**Sex**			
Male	5,202	38.1 (36.5–39.8)	1,116 (331–2,886)
Female	6,486	40.8 (39.3–42.3)	1,047 (320–3,097)
**Age group (yrs)**			
18–24	1,033	34.4 (31.1–37.9)	332 (122–730)
25–34	2,224	54.4 (52.1–56.7)	657 (248–1,645)
35–44	2,214	55.1 (52.7–57.5)	1,403 (384–3,428)
45–64	4,154	35.9 (34.2–37.7)	2,235 (645–5,105)
≥65	2,032	13.1 (11.5–14.7)	2,332 (614–5,613)
**Race/Ethnicity**			
White, non-Hispanic	8,153	35.2 (33.9–36.5)	1,545 (454–3,757)
Black, non-Hispanic	1,668	57.4 (54.5–60.3)	534 (192–1,575)
Hispanic/Latino	1,371	47.1 (43.5–50.8)	792 (290–2,092)
Other, non-Hispanic	496	31.3 (26.5–36.4)	702 (173–1,903)
**Education**			
<High school	1,619	40.1 (37.3–43.1)	844 (273–2,290)
High school	3,172	34.6 (32.5–36.7)	1,033 (285–3,241)
Some college	3,184	44.6 (42.5–46.7)	954 (301–2,736)
College or above	3,701	39.2 (37.3–41.2)	1,388 (425–3,592)
**Annual household income**			
<$35,000	4,084	44.1 (42.3–46.0)	767 (260–2,236)
≥$35,000	6,553	39.1 (37.6–40.6)	1,356 (391–3,448)
**U.S. census region**			
Northeast	1,939	38.3 (35.5–41.1)	862 (292–2,901)
Midwest	2,751	32.9 (30.7–35.1)	1,203 (356–3,461)
South	4,281	41.8 (39.9–43.7)	998 (305–2,859)
West	2,717	43.2 (40.6–45.9)	1,226 (351–3,112)
Recent HIV risk*			
Yes	1,693	62.2 (59.2–65.1)	512 (172–1,357)
No	9,995	36.1 (34.9–37.3)	1,360 (401–3,510)

**Abbreviations:** CI = confidence interval; IQR = interquartile range.

* Had male sex partner (male respondents only), had multiple sex partners, injected drugs, paid or was paid for sex in past 12 months.

**FIGURE:**
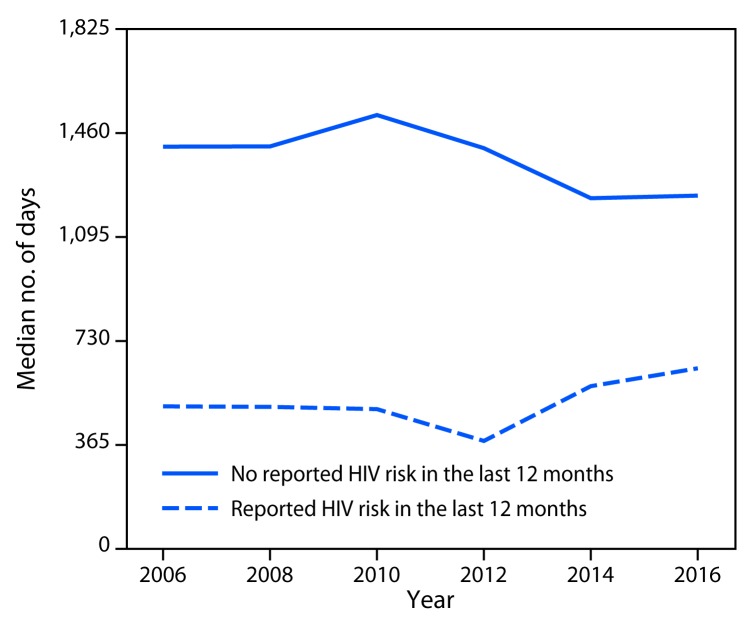
Median interval in days since last HIV test among men and women with and without recent HIV risk in past 12 months, by survey year — General Social Survey, United States, 2006–2016 **Abbreviation:** HIV = human immunodeficiency virus.

**TABLE 2:** Percentage of persons tested for human immunodeficiency virus (HIV) infection and median number of days since last HIV test, by HIV risk group, General Social Survey — United States, 2006–2016

HIV risk group*	No. (%)	Ever tested	Tested in past 12 months	No. of days since last test
		Weighted % (95% CI)	Weighted % (95% CI)	Median (IQR)
Men with recent male sex partner	180 (1.5)	71.0 (62.1–78.5)	42.2 (32.7–52.4)	459 (172–2,143)
Men with other recent risk	849 (7.3)	58.0 (53.9–61.9)	37.0 (31.7–42.7)	610 (202–1,434)
Women with recent risk	664 (5.7)	65.9 (61.1–70.4)	45.6 (39.9–51.4)	416 (139–1,169)
Men and women with no recent risk	9,995 (85.5)	36.1 (34.9–37.3)	23.6 (22.0–25.3)	1,360 (400–3,510)

**Abbreviations:** CI = confidence interval; IQR = interquartile range.

* Recent risk includes having a male sex partner (male respondents only), having multiple sex partners, injecting drugs, and paying or being paid for sex in the past 12 months. Risk groups are mutually exclusive. Male respondents with a male sex partner were classified as having a male sex partner regardless of any additional reported risks.

## Discussion

In this analysis, the median estimated interval since last HIV test for persons with recent HIV risk was 512 days (1.4 years). Although persons with recent HIV risk were more likely to have ever tested and to have tested more recently than those without recent risk, during 2006–2016 the median estimated interval since last test remained consistently longer than 1 year for all three risk groups defined in this analysis. Although longer than annual screening, the median estimated interval since last test was shorter among women with recent risk than among men with recent risk; this likely reflects the contribution of prenatal screening, which is commonly reported as the main reason for testing among women (*8*). These findings suggest that persons with HIV risk in the past year are not testing as frequently as recommended, consistent with findings from NHSS, which reported that the median interval from infection to diagnosis was ≥2 years for all risk groups (*2*). NHBS data from the same report indicated that 71% of MSM but only 41% of heterosexual men and women had tested in the past year. In this analysis, the percentage of all groups with recent HIV risk who tested in the past year was less than 50%, which is comparable to testing estimates among MSM sampled by other population-based surveys such as BRFSS (*9*) and the National Survey of Family Growth (*8*) as well as national web-based surveys of MSM (*10*). GSS is the only national population-based survey that provides enough risk information to stratify testing estimates by HIV risk while also providing single-year testing estimates.

The findings in this report are subject to at least four limitations. First, because the proportion of respondents reporting specific HIV-related risk behaviors in the past 12 months (e.g., injecting drugs) was small, trends in the interval since last test could not be evaluated by individual risk group, which could have obscured meaningful differences between risk groups. Second, self-reported data might be compromised by social desirability and recall biases, which might have led to overestimates of testing among persons with HIV risk. Third, because GSS is a household-based survey, important subgroups of persons with recent HIV risk, such as persons who inject drugs or homeless persons, were likely undersampled. Finally, to the extent that those who answered “don’t know” or refused to answer the HIV testing question were at higher risk for HIV infection and were not being tested frequently, the median interval since last test among persons at risk could have been underestimated.

Early diagnosis and effective treatment that suppresses HIV replication not only reduces individual morbidity and mortality but also reduces the risk for transmission to others.^¶^ Delayed diagnosis limits the benefits of early treatment initiation to minimize immune system damage and prevent HIV transmission. HIV screening is a critical entry point to a range of HIV prevention and treatment options. For persons with ongoing risk for HIV infection, annual screening also offers the opportunity to discuss options to reduce risk, including HIV preexposure prophylaxis.** Findings from this analysis suggest that HIV screening frequency for persons with recent HIV risk is suboptimal and has not improved substantially since 2006. Continuing efforts are needed to achieve full implementation of annual screening recommendations and prevent new infections. It is important that health care providers and public health practitioners intensify efforts to identify persons with ongoing risk and ensure they are engaged in annual screening for HIV infection. Strategies that have been shown to be effective for increasing one-time screening, such as integration of routine screening as standard clinical practice through supportive institutional policy changes, electronic health record prompts, and staff member education (*3*) could be used to ensure repeat screening for persons with ongoing risk. Expanding access to HIV screening in nonclinical settings and through strategies such as social network strategy, couples HIV testing and counseling,^††^ and home testing^§§^ can reduce barriers to accessing screening. New, innovative approaches might also be needed to increase repeat screening of persons with ongoing risk.

**Summary**

**What is already known about this topic?**

CDC recommends routine human immunodeficiency virus (HIV) screening of persons aged 13–64 years and annual rescreening of persons at higher risk. Many persons at higher risk are not screened annually.

**What is added by this report?**

Analysis of 2006–2016 national population-based data found that the percentage of persons ever tested and median interval since last test remained unchanged. The median interval since last test among persons with recent HIV risk was shorter than that of other persons tested but exceeded 1 year.

**What are the implications for public health practice?**

Efforts to identify persons at higher risk and ensure that they receive annual HIV screening can reduce morbidity, mortality, and transmission to others. Integration of routine screening as standard clinical practice through existing strategies, such as electronic medical record prompts, or new, innovative strategies might be needed to increase repeat screening of persons with ongoing risk.

## Footnotes

* Conducted by the National Opinion Research Center at the University of Chicago. http://www.gss.norc.org/.

† Time since last HIV test was assessed during the interview by asking respondents for the month and year of their last test. In this analysis, the number of days since last test was estimated by randomly assigning a day in the month of last test and subtracting that date from the date of interview. If the respondent was last tested in the same month as the interview, the number of days since last test was randomly assigned to between 1 and 31 days.

§ http://www.gss.norc.org/documents/codebook/GSS_Codebook_AppendixA.pdf.

¶ https://www.cdc.gov/hiv/risk/art/index.html.

** https://www.cdc.gov/hiv/risk/prep/index.html.

†† https://effectiveinterventions.cdc.gov/en/HighImpactPrevention/PublicHealthStrategies/HIVTesting.aspx.

§§ https://www.cdc.gov/hiv/testing/hometests.html.
